# “It’s Easy to Put Oneself in the Shoes of Others.” Results of a School Study in Geography Lessons on Working with Authentic Personal Narratives in Comparison to Factual Texts

**DOI:** 10.3390/ejihpe13060081

**Published:** 2023-06-18

**Authors:** Astrid Lütje, Alexandra Budke

**Affiliations:** Institute for Geography Education, University of Cologne, Gronewaldstraße 2, 50931 Cologne, Germany; alexandra.budke@uni-koeln.de

**Keywords:** narrative, factual text, authenticity, multi-perspectivity, memory, motivation

## Abstract

Texts represent the most frequently used medium in geography teaching, although they do not belong to leading subject-specific media. Despite their undisputed didactic importance, they have not yet been sufficiently researched. In this article, we consider the use of authentic and personal narratives in geography lessons. We first show their theoretical potential for realistic, multi-perspective and motivating teaching. Then, we present a school study in which the use of authentic, personal narratives was investigated in comparison to a factual text. The areas of investigation were the students’ understanding of the content of a geographical topic, their memory performance and their motivation to work. The results show that authentic, personal narratives are better suited than factual texts to convey a topic to pupils in a multi-perspective and differentiated way. They also confirm their potential to empathise better with other people and to understand their actions through changes in perspective. Regarding recall performance, however, the results show no difference between the two groups. Finally, the results of the school study are considered in the context of forming suggestions for the use of authentic, personal narratives in geography lessons.

## 1. Introduction—Texts in Geography Lessons

Since the development of the teaching/learning theoretical approach by the Berlin School in the 1970s, the use of media in teaching has been regarded as an independent didactic decision field [1]. It is closely correlated with the three other decision fields of lesson planning: intentions and goals of the lesson, topics and contents, and methods and procedures. As a fourth element, media are essential for effective teaching.

After the spoken word, texts are one of the oldest means of communication in teaching. In contrast to maps and remote sensing images, whilst texts do not belong to leading subject-specific media in geography didactics, they nevertheless represent the most frequently used medium in teaching [2] (p. 187), [3] (p. 182), [4] (p. 343), [5] (p. 308). In general, media substitute for direct encounters with realities that cannot be experienced in class due to spatial or temporal distance. Since geography lessons often deal with phenomena and problems that are not "on the school’s doorstep" and, thus, do not enable direct experience, the use of media, including text, is vitally important in geography lessons [6] (p. 268). 

With regard to the provenance of texts, a distinction is made between didactic texts used in the classroom, which are written specifically for school purposes (especially in textbooks and on worksheets), and authentic texts. The latter include all texts published in non-school contexts, such as newspaper and magazine articles, as well as text extracts from books and digital media [2] (p. 187), [4] (p. 344). In terms of content, a distinction is made between the two large text groups of fiction and non-fiction. Non-fiction refers to a reality outside the individual’s world of imagination.

Non-fictional texts are used almost exclusively in geography lessons, but they represent a heterogeneous group due to their great stylistic variance. This type of texts is mostly written by textbook authors or teachers; extracurricular source texts are also used. Two fundamentally different groups of texts can be distinguished: Factual texts convey their content concisely, clearly and with the greatest possible concentration on the essentials. Personal narratives, on the other hand, are characterised by a vivid, embellished and often exciting narrative style. They, too, report facts, but in a personal and often emotional way. The first group includes descriptions, reports, explanations and interpretations, while narratives of experiences or narratives of travel are examples that belong to the second group [4] (p. 342).

The traditional view that personal narratives are more suitable for lower and middle school students while factual texts are more suitable for upper school students is now being questioned. Even if an age-specific appropriate form of presentation has to be chosen, it does not mean that personal narratives should be reserved for younger pupils. This is particularly the case in the context of inquiry-based learning, as media formulated experientially can be used effectively in upper schooling. Through such texts, geography lessons can become an experiential space in which questions arise and answers are not passively received, but productively sought through one’s own efforts [7] (pp. 45–47). 

Previous research on the texts used in geography textbooks covers a variety of aspects such as content, language, pedagogical style and design [8,9]. The question of which geographical topics should be selected and which places should be represented in a geography textbook is debated in different countries. Racism and other forms of discrimination as well as stereotypes and prejudices have been highlighted for several decades [10,11,12]. Furthermore, the constructivist perception of the world with its focus on knowledge production has led to an increased demand for a multi-perspective representation of geographical topics [13,14,15]. Recently, political aspects have gained importance in the literature on geography education [16,17]. The impact of language and design on the learning process and learning outcomes is also one of the main topics in academic research, e.g., [18,19,20]. However, the impact of different types of texts on the learning process has not been investigated so far. As such, this study aims to address this research gap.

In this article, we consider the use of authentic and personal narratives in geography lessons. We first show their theoretical potential for realistic, multi-perspective and motivating teaching. Then, we present a school study in the German context in which the use of authentic and personal narratives was investigated in comparison to a factual text, using the example of internal migration in Egypt. Three questions guided the conception and evaluation of the school study:

- To what extent do students acquire a more differentiated understanding of geographical topics by working with personal narratives compared to working with factual texts?
- To what extent do geographical contents remain better in memory when they are acquired through personal narratives compared to a factual text?
- Are students more motivated when working with personal narratives compared to working with factual texts?

Finally, the results of the school study are considered in the content of forming suggestions for the use of authentic, personal narratives in geography lessons. 

## 2. Theoretical Foundations

### 2.1. The Problem of Using Factual Texts from the Geography Textbook in the Classroom

An essential part of written media that pupils are confronted with in their geography lessons consists of the factual texts in geography textbooks. Their characteristic features include the concise presentation of content, which is limited to the essentials, a clear and precise way of expression and a stringent sequence of thoughts, which is reflected in the external structure. In this, these textbooks follow the “Hamburger Verständlichkeitskonzept” (Hamburg comprehensibility concept), which highlights four aspects that optimise the comprehensibility of a text: linguistic simplicity, clear internal structure and clear external outline, conciseness of content and motivating text elements [21]. Factual texts describe, analyse, interpret and explain events and facts. The type of presentation is object-related, i.e., personal opinions and assessments must be marked as such for the reader [4] (p. 342).

These texts can be used effectively in geography lessons because they are written in a way that is relevant to the target group and the content is presented in a condensed form. Because of their small size, they are also very suitable for the double-page principle of German textbooks. However, the more complex language of explanatory texts compared to everyday language can lead to comprehension difficulties, particularly for younger children [19]. Furthermore, even if factual texts “attempt to convey reality in as objective a form as possible”, this claim can never be fulfilled [22] (p. 236). This is because every factual text only reproduces a specific perspective on reality and, as such, only presents a selection of aspects taken from scientific research results and compiled under the precept of didactic reduction. The authors decide which perspectives and aspects are chosen and, therefore, incorporate ideological principles and the orientation towards certain values into the text design, either consciously or unconsciously. As a result, factual texts can never reproduce an “objective” reality, but instead construct a reality. Studies have shown that geography textbooks do not present a multi-perspective and balanced picture of geographical regions and issues. The predominant perspective is that of the authors. Most of the topics presented in textbooks are viewed from the perspective of an academic rather than from the perspective of a person directly affected. In most cases, therefore, the diversity of perspectives is not represented [10,13,14,15]. However, experience shows that textbook texts are often unquestioningly accepted and reproduced by students as "objective truth". A critical reflective approach that raises awareness of the conditions under which they were created is probably rarely encountered in class [22] (p. 236), [23] (p. 140). 

An alternative to textbook texts is given in the use of authentic texts that express different perspectives on a topic. They include reports, descriptions and explanations, and personal narratives and statements, which can be supplemented by non-textual media. One could argue that personal narratives are individual cases and, therefore, hardly suitable for an overall understanding of a topic. However, this view can be countered by the fact that within the framework of qualitative methods in the social sciences, autobiographical narrative interviews have been generally recognised as a medium for gaining knowledge since the 1980s [24,25]. If one understands teaching as a place of independent research-based learning, then personal narratives can play a role in giving insight into a topic. 

### 2.2. The Potential of Personal Narratives in Geography Lessons

Of the two aforementioned groups of authentic texts—factual reports, descriptions and explanations on the one hand and personal narratives on the other—the potential of the latter for geography lessons is considered in detail with regard to subject-related and didactic aspects. In contrast to factual texts, personal narratives also express personal feelings, sensations and thoughts that are connected to the narrated event [26] (p. 265–269). They open up a level of encounter with reality that is not possible through factual texts. For this reason, we would like to point out that personal narratives are well suited to the achievement of three basic objectives in the content of geography teaching:

1. To clarify the subject-bound nature of any experience of the world;
2. To recognise emotions as a constitutive part of world perception;
3. To promote the understanding that knowledge of the actors’ intentions is fundamental to understanding spatial processes.

With reference to the literature on the didactics of geography, these three objectives are now explained in more detail. 

Objective 1: The personal formulation of the aforementioned text types indicates that every encounter with reality is a subject-related experience and, in the understanding of constructivism, a subjectively constructed reality, which does not claim to reflect its content “objectively and truly”. Among geography didacticians, it is Rhode-Jüchtern, among others, who developed the constructivist understanding of reality into a conceptual guideline for teaching materials and their use in teaching. He considered authentic and personal narratives as the central medium of a geography lesson, in which space is represented by a selection of perspectives of its actors. Learners perceive their narratives, interpret them in their socio-cultural context, and supplement them with further narratives and facts [27] (pp. 68–80), [15] (pp. 126–142), [28] (pp. 49–61). Perspectivity raises awareness of the perspective-bound nature of any perception of the world, including one’s own. Even space-related conflicts can only be understood in their deep structure by reflecting on the perspective-boundedness of their actors. 

Objective 2: Personal narratives do not exclusively serve to convey knowledge to be acquired cognitively, at the same time providing the opportunity to feel conveyed reality. The readers are addressed emotionally; they develop feelings and sensations that can be made conscious through appropriate teaching tasks. This creates fertile ground for the development of empathy and for the acquisition of the ability to change perspectives [26,29,30,31,32,33].

Objective 3: With the increased focus on personal narratives, geography teaching can turn away from the priority of describing and explaining space and instead place the forces that shape space, i.e., human activities with their respective socio-cultural background, at the centre of the world encounter taught. This turn from the observation of space to the recording of its shaping actors has been successively carried out in human geography in recent decades [34,35,36,37,38].

Teaching with personal narratives can also serve didactic purposes, two of which are considered in more detail in the context of more recent psychological research:

4. Strengthening the ability to remember;
5. Increasing work motivation.

Objective 4: In learning psychology, memory formation is compared to a storage process. According to this model, information processing can be divided into the three steps of reception, storage and retrieval. However, only a very small part of the stimuli received reaches the short-term memory as a result of conscious attention. From there, they are transferred to long-term memory or they are lost [38]. The interesting question for teaching is how to prevent important content from being lost on the way to long-term memory. Recent research has shown that information processing carried out independently by students has a positive effect on memory performance. This can happen, for example, in an independently worked out summary of the learning content or in a transfer performance. Passive memorisation is replaced by active engagement with the content and its independent reconstruction. The more thoroughly this process is undertaken, the easier it is to memorise the content in the long-term memory [39] (pp. 58–71). 

In view of these contexts, it is to be expected that the textbook’s factual texts, which are designed according to didactic aspects and have an apparent perfection, tempt students to learn them off by heart. Working with authentic personal narratives, on the other hand, requires independent and active engagement, which can support long-term memorisation. 

Objective 5: Among the didactic potentials is also the positive effect of working with personal narratives on work motivation. Several studies have shown that there are essentially three basic needs in relation to learning, the satisfaction of which determines whether a behaviour is intrinsically motivated: self-determination in the design of the learning and working process; experience of competence during task processing; and social integration in the learning group [40] (pp. 102–121). 

The three needs are taken into account when working with personal narratives. Since these texts always express individual perspectives, they must be supplemented with texts from other perspectives and, if necessary, with other materials in order to achieve the most comprehensive understanding of the subject matter being discussed. The different levels of difficulty of the texts give teachers the opportunity to take into account the different abilities and performance levels of the pupils in a lesson, which enables each pupil to have an individual experience of competence. There are also many opportunities for cooperative learning and the development of social skills. Ideally, this can lead to lessons that are experienced as being motivational by the learners, which are, therefore, not only “fun” but also lead to a long-lasting, deeper understanding of the content. 

Although working with personal narratives in geography lessons can be well justified by the aforementioned subject-specific, didactic and learning–psychological research, they are rarely used in practice. This was shown by an analysis of 30 secondary-level geography textbooks from North Rhine-Westphalia conducted by the authors [41,42]. Personal narratives occurred on only 6.2% of all chapter pages. In geography textbooks for 10- to 12-year-olds, personal narratives make up 10.4% of the content, but this decreases to 6.5% for 14- to 16-year-olds, and only 2.0% for 17- to 19-year-olds. Moreover, the majority of these texts are not authentic, but rather invented by the textbook authors, with only 14.4% of all the personal narratives examined containing a source reference [41] (pp. 40–41). Multi-perspectivity occurs in 63% of all personal narratives, but a small selection of perspectives and often inadequate contextual information are included overall [42] (pp. 51–57). 

The textbook analysis also revealed that the vast majority of personalised texts examined do not contain emotions, instead conveying facts, explaining contexts and expressing value judgements. In only 14.4% of the texts do the speakers also express feelings and sensations. The majority of the personal narratives are, thus, “disguised factual texts”, since the personal way of presentation has no relevance to the content but is to be evaluated as a motivational element [41] (p. 43).

## 3. Materials and Methods

The objective of the school study was to find out whether learners at the lower secondary level understand a geographical topic in a more differentiated way, can retain it in their memory for a longer period of time, and consider the tasks to be more motivational when they are confronted with authentic, personal narratives compared to working with factual texts. Internal migration in Egypt was chosen as the content topic.

### 3.1. The Subject of Internal Migration

Internal migration and the associated urbanisation processes are phenomena that can be observed worldwide. These topics are also firmly anchored in the curricula of German geography lessons. In view of the global topicality and due to their relevance for teaching, these subject areas were considered to be suitable for conducting a didactic school study. 

The text material presented to the students dealt with the causes and effects of internal migration in Egypt. It included various reasons why inhabitants of rural areas had left their home villages and moved to the capital Cairo. The consequences of Cairo’s rapidly growing population were also addressed. The choice of Cairo as an exemplary place for internal migration and urbanisation was due to the personal connection of one of the authors (AL) with this city, where she lived and worked for several years and, thus, had the opportunity to get to know the country from the perspective of locals. 

Although Egypt is a suitable example of internal migration and urbanisation, it must be taken into account that the situation in Egypt cannot be transferred to other geographical areas without adaptation. The reasons that cause and promote internal migration are different in each country depending on the respective natural geographic, economic, social and political conditions. 

### 3.2. Criteria for the Selection of Text Material

Due to the comparative conception of the school study, not only were several authentic, personal narratives on internal migration in Egypt needed, but also a factual text summarising these contents. It was only if the texts were constructed in this way that it could be ensured that all students were taught the same factual knowledge through the reading of their text material, regardless of whether they worked with the personal narratives or with the factual text. The factual text can be found in the supplementary materials (Supplementary: Factual text); for the personal narratives, see ref. [43].

The personal narratives used are excerpts from interviews that were either published in English-language Egyptian print or online media in Cairo or conducted by the author (AL) herself from 2013 to 2014. The selection was guided by the principle of multi-perspectivity. The Egyptians whose perspectives are presented as examples represent different social groups within contemporary Egyptian society in terms of their place of residence (rural areas or Cairo) as well as their age, gender and social background. The statements, thus, do not merely represent different individual opinions but are also representative of certain social groups. As such, the selection of texts was challenging. 

More than 20 years ago, Rhode-Jüchtern developed the model of the perspective cube, whose three dimensions provide clear methodological suggestions for perspective selection [27,44]. Based on this model, we chose the following criteria for our perspective selection:

- Professional perspectives: economy, urban development, transport, living together in society, education;
- Perspectives of certain social groups formed on the basis of the following criteria: place of residence (rural or urban area), age, gender, social origin (class affiliation);
- Individual perspectives: personal life plans and perspectives.

A complete representation of the diversity of perspectives within Egyptian society cannot be achieved. Thus, each selection is only an approximation of complete representation. The following table (Table 1) illustrates the extent to which the three criteria are represented in our selection of texts: 

### 3.3. Design and Implementation of the School Study

**Figure 1:**
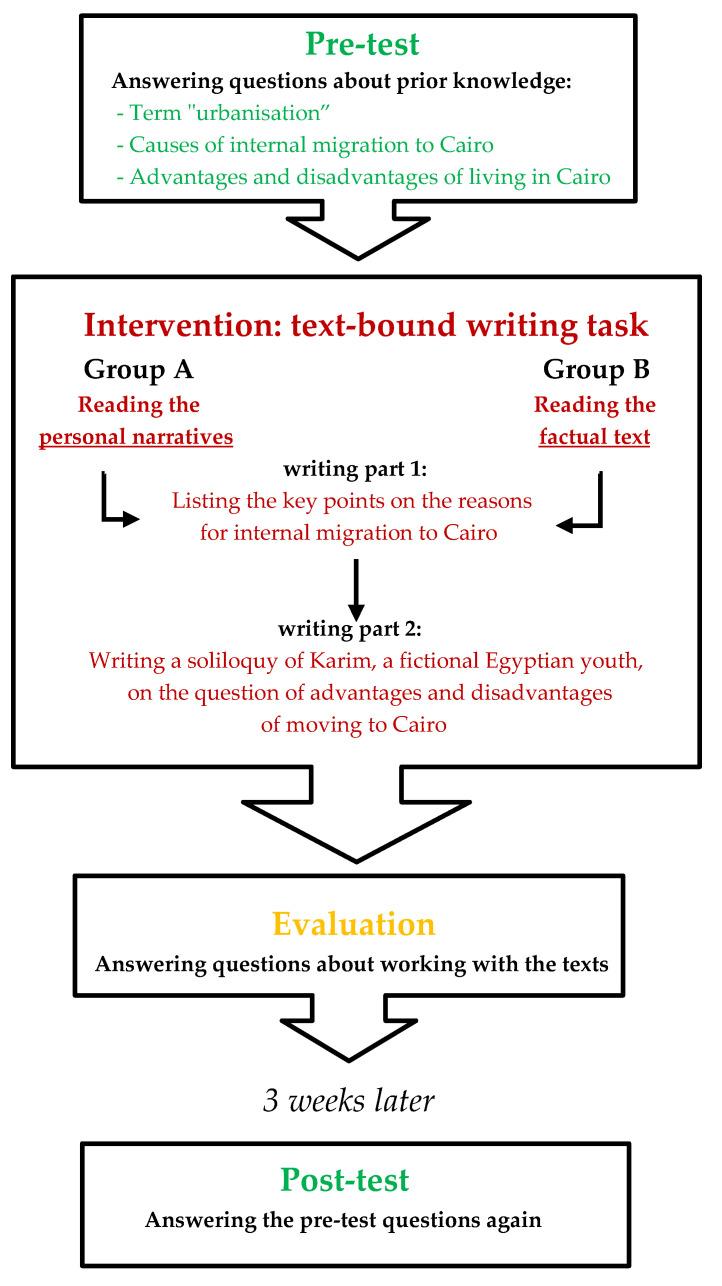
Schematic representation of the sequence of tasks in the school study.

The school study was conducted in January 2022 at a gymnasium in North Rhine-Westphalia, Germany, in two parallel grade-9 classes. The students were 14–15 years old. The study consists of three parts—pre-test, intervention in the form of a text-based writing task with evaluation, and post-test—and was realised within the framework of two timed lessons of 70 min each. Thirty-six students took part: 16 girls and 20 boys. The data collection was anonymous. A code, which was created by the students themselves according to certain guidelines and noted on all the task sheets, enabled us to correctly assign the task sheets. The following diagram (see Figure 1) visualises the sequence of tasks that the students had to complete:

The questions in the pre-test relating to prior knowledge are identical to the questions in the post-test: 

Question 1: What does the term “urbanisation” mean? 

Question 2: Why do people in Egypt leave their home villages to live in Greater Cairo? 

Question 3: What advantages does living in Greater Cairo have for people from the rural areas of Egypt?

Question 4: What are the disadvantages of living in Greater Cairo for the people from the rural areas of Egypt?

With the help of the pre-test, we were able to determine the students’ prior knowledge on the topics of urbanisation and internal migration in Egypt before reading the text material. The results of the writing task and the post-test were later compared with the prior knowledge and evaluated. The topics of urbanisation and internal migration, and North Africa in general, had not yet been covered in detail in class. The post-test aimed to find out how much of the knowledge acquired through reading and working on the text material could still be remembered three weeks after working with the texts. The three-week interval between the intervention and the post-test resulted from our research design, which is based on a short-term intervention. The literature does not specify fixed time periods for intervention studies. The time interval should be neither too short nor too long to allow for evidence of change [45]. For our study, a period of three weeks seemed appropriate.

Following the pre-test, the text material and the writing task were distributed. In order to create a comparative situation necessary for later evaluation, about half of the students in both classes received the personal narratives (group A), while the other half worked on the factual text (group B). Groups A and B were formed through random selection by drawing lots. Due to its narrative character, the text material for group A is longer than the factual text used for group B (1590 vs. 949 words). However, since the processing time of the writing task was ample, this difference was not detrimental. The task is the same for both groups and consists of an introduction (Box 1) and two subtasks (Box 2):

Box 1 **Introductory text to the writing task.**

 **Introduction:** You travel to Egypt for a fortnight with a tour group. Your Egyptian guide not only takes you to the sights, but also organises a visit to his home village in the Nile Delta. His father owns his own land there, where he grows fodder clover for his animals and vegetables that he sells at the market. His mother takes care of the large household and the four children. The two oldest children, your guide and his younger brother Karim, already earn money and contribute to the family income. The two youngest children are still at school. The family is not rich, but they have everything they need to live: a small house of their own and enough money for their daily needs. The parents are happy that they were even able to finance the eldest son’s studies to learn foreign languages. As a result, he can now work in tourism.

 Your guide introduces you to his 17-year-old brother Karim. You like Karim and want to ask him some questions about life in Egypt. The guide offers to interpret. Karim tells you that he went to school for nine years and finished with a secondary school diploma. Now he has been helping his father in agriculture for two years and earns additional money by working on construction sites. However, he is thinking of going to Cairo and building his own life there. This step would have advantages compared to his current living situation, but also disadvantages. Hani, one of his best friends, likes the idea of living in Cairo and would come with him. However, another friend, Youssef, advises him against it and asks him to stay in the village.

Box 2 **The writing tasks.**

 **Tasks:** Now read the personal narratives (group A) or the factual text (group B) and then answer the following two tasks:

1. List the reasons why people in Egypt are leaving their villages and settling in the capital region of Greater Cairo using a number of key points.

2. Now put yourself in Karim’s situation. Present Karim’s personal point of view in the form of self-talk: Should he stay in the village or move to Cairo? Take into account as many aspects as possible and justify your considerations with realistic arguments. Conclude with a decision.

Karim writes (continue the text):

 *Today I thought again whether I should stay here in the village or whether I’d rather go to Cairo....*

The first part of the writing task (the list of key points) serves to prepare content for the second writing task (self-talk). In order to facilitate the change of perspective necessary for the formulation of the self-talk, some imaginative aids about Karim’s life were given in the introduction: place of residence; family situation; education; work situation; financial possibilities. 

The final post-test took place three weeks after the writing task. The students were asked to answer the questions from the pre-test again. The final planned discussion, which was supposed to be a reflection on the work process and to summarise the knowledge gained, unfortunately had to be cancelled due to the school’s internal COVID-19 rules. 

### 3.4. Methods of Analysing the Students’ Texts

For establishing the differences between the two groups, descriptive data methods were applied. The analysis of the empirical material obtained from the pre-tests, writing tasks and post-tests is guided by three research questions, which are explained below. 

**Research question 1:** To what extent do students acquire a differentiated understanding of geographical topics by working with personal narratives compared to working with factual texts?

The question can be divided into four sub-questions with detailed reference to our school study (group A worked with the personal narratives and group B with the factual text): 

**Sub-question 1.1.:** Are the students’ texts of part 2 of the writing task (Karim’s self-talk) more extensive in group A than in group B? 

To answer sub-question 1.1., we calculated the average number of words used by the students in Karim’s self-talk for both groups A and B. Afterwards, the calculated average values were compared with each other. 

**Sub-question 1.2.:** In the two parts of the writing task, do the students in group A give more appropriate reasons for or against moving to Cairo than the comparison group B? 

To address sub-question 1.2., we examined two aspects: First, we asked whether changes in the students’ knowledge of the causes of Egyptian internal migration could be determined as a result of the text readings, and to what extent these changes differed between groups A and B. For this purpose, we determined the average number of reasons given for this question for group A and group B from the pre-tests. We then analysed the results of part 1 of the writing task in the same way. Part 1 asked the students to name the reasons for internal migration as a series of key points immediately after reading the text material. The average scores of the two groups could finally be compared and interpreted with regard to the first research question.

Furthermore, we identified and categorised the number of valid reasons that the fictional Karim gives for or against moving from his village to Cairo from the answers to the writing task from part 2 (Karim’s self-talk). The following text example (see Table 2) illustrates our procedure for categorising the reasons (all student texts in this article have been translated into English by the authors.): 

This text example in Table 2 was of average length compared to all the students’ texts and was structured exactly to the specifications of the writing task. After the introductory sentence, the student listed seven reasons, four of which were for moving to Cairo and three against. Finally, she came to a decision. All reasons are based on information from the text material. The first six reasons corresponded to the real living conditions and opportunities in Cairo and can, therefore, be considered appropriate reasons. However, whilst the last reason reflects the problem of mobility in Cairo it does not take into account the higher costs of owning a car compared to using public transport. Consequently, the lack of background knowledge on the part of the student led to an inappropriate reason, which, therefore, had to be classified as a misconception.

For part 2 of the writing task, the average number of appropriate reasons used was calculated for both groups A and B separately. The results were then compared and interpreted with regard to research question 1.

**Sub-question 1.3.:** Are there any differences in the content of “Karim’s self-talk” between groups A and B with regard to the use of appropriate reasons and misconceptions?

After analysing the appropriate reasons used by the students in Karim’s self-talk, the average number of misconceptions was determined. By comparing the two groups, we wanted to find out whether there were differences regarding the number of appropriate reasons and misconceptions. The different text material used in each group could be the reason if this is the case. This assumption leads to sub-question 1.4. 

**Sub-question 1.4.:** To what extent are these differences based on the text material used in each group?

In order to answer the last sub-question 1.4, all student texts (“Karims self-talk”) were analysed with regard to the origin of the reasons used. The students used a total of three different sources:

- The text material (personal narratives or factual text);
- The introductory text to the writing task with background information on Karim’s life situation (identical for both groups);
- Reasons independently developed by the students, originating from their own life situation and transferred to Karim’s life in a more or less modified form.

In the text example presented (see Table 2), the information underlying reasons 2, 3, 4, 5 and 7 is sourced from the text material (see the right column “text-source of reasons” in Table 2). The knowledge of reasons 1 and 6 is taken from the introductory text to the writing task. The seventh reason was an independently developed, though not appropriate, aspect with reference to the costs of bus journeys. This was also the case for the final sentence, which still deferred the decision. After identifying the sources, the category-specific number of sources used was determined for each student and then their average value was calculated for the two groups. 

**Research question 2:** To what extent is geographical knowledge better remembered when acquired through personal narratives compared to factual texts?

For this purpose, we compared the results from part 1 of the writing task (the key point list of reasons for internal migration to Cairo) with the results of the same question of the post-test, which was conducted three weeks after the writing task. First, we determined the average number of appropriate reasons mentioned in the initial list of reasons and for the corresponding question of the post-test, as well as the misconceptions for group A and group B. Then the results of the two groups were compared with regard to the second research question.

**Research question 3:** Are students more motivated when working with personal narratives compared to working with factual texts?

In order to determine the differences in motivation between groups A and B, we analysed the polarity table and the students’ written comments, which were written immediately after the writing task. The guiding question was whether the students in group A gave more positive feedback than the comparison group B. 

The polarity table (see Table 3) is designed in the form of a Likert scale and contains six aspects on which the students were to assess the text material used. To enable quantitative analysis, each aspect was given a certain number of evaluation points (ascending from one point in the right column to seven points in the left column). This was supplemented by the qualitative analysis of the personal comments on working with the text material.

## 4. Results

### 4.1. Differentiated Text Comprehension

#### 4.1.1. Scope of the Students’ Texts

**Figure 2:**
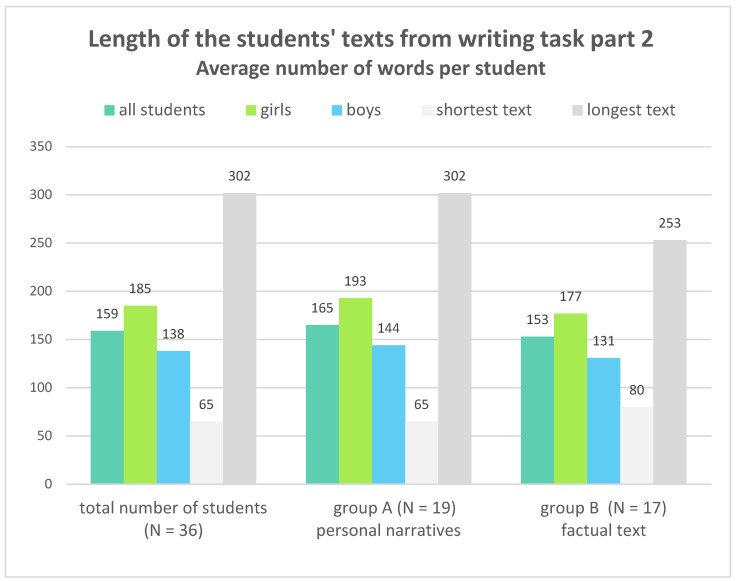
Length of the students’ texts from writing task part 2—average number of words per student, differentiated by text material and by gender (authors’ diagram).

First, we examined the students’ texts from part 2 of the writing task (Karim’s self-talk) with regard to their length (see Figure 2). The average number of words in all 36 students’ texts was 159, with the girls being about 16% above the average and the boys about 13% below. However, the range of text lengths were very varied, with the longest text being 302 words and the shortest only 65. The texts written by group A, which were based on the personal narratives, were on average 7.8% longer than the students’ texts of group B, which were written on the basis of the factual text. However, this difference is not sufficient to convincingly prove that group A has a larger text volume in the sense of research sub-question 1.1.

#### 4.1.2. Increase in Knowledge about the Topic

**Figure 3:**
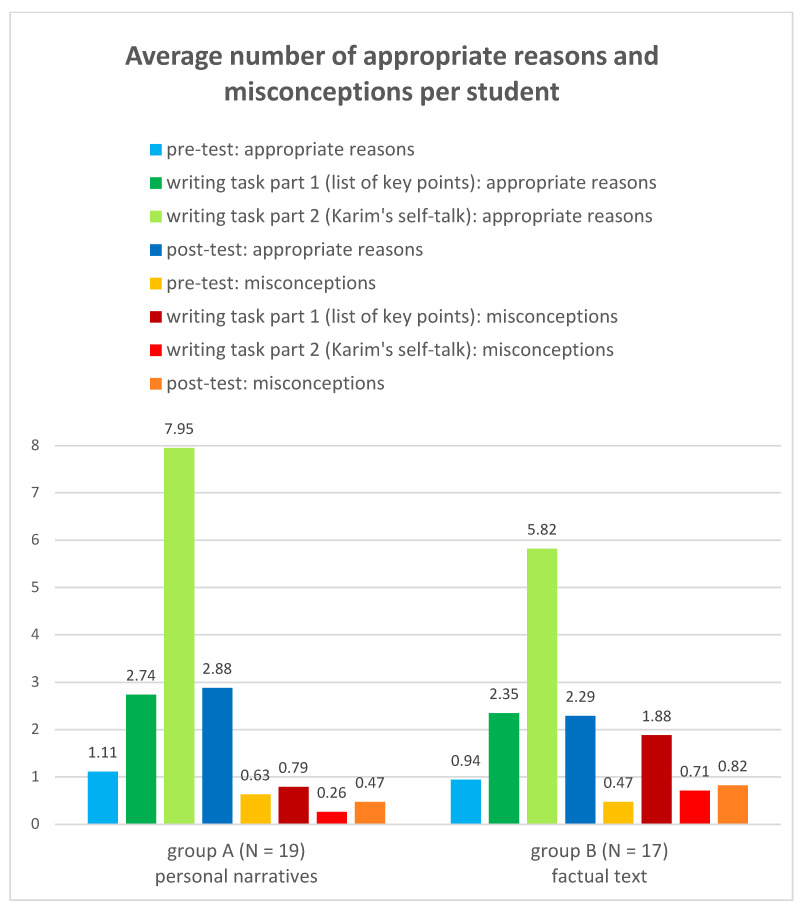
Average number of appropriate reasons and of misconceptions given per student in the pre-test, in the writing task and in the post-test (authors’ diagram).

In connection with the second sub-question 1.2., we tried to find out whether and to what extent the students’ level of knowledge changed as a result of reading the text material provided (personal narratives and factual text). To do this, we first compared the prior knowledge about the reasons for internal migration to Cairo, documented in the second question of the pre-test, with the key point list of reasons created by the students immediately after reading the text material (writing task, part 1). A distinction was made between appropriate reasons and misconceptions. The results are shown in Figure 3. These results show that knowledge of the reasons for internal migration increased by around 150% compared to the pre-test as a result of working with the text material in both groups. In group A, the number of appropriate reasons increases from 1.11 (pre-test) to 2.74 (key point list) and in group B from 0.94 to 2.35. It is, thus, clear from the data that both the personal narratives and the factual text are equally suitable for conveying factual knowledge. 

We then identified the number of reasons and counter-reasons given for or against moving to Cairo in the second part of the writing task (Karim’s self-talk). Since reasons against internal migration could also be given in this task, the total number of reasons and counter-reasons given is significantly higher compared to the pre-test and the list of key points. The number of appropriate reasons given in group A exceeded the number of appropriate reasons given in group B by more than a third (37%). Students in group A gave an average of 7.95 appropriate reasons, while in group B only 5.82 appropriate reasons were given. Sub-question 1.2. can, therefore, be answered affirmatively without reservation. It can be assumed that the vivid style of the personal narratives is better suited to empathising with Karim’s life situation and for developing realistic reasons for or against the move to Cairo.

#### 4.1.3. Appropriate Ideas and Misconceptions

In order to answer the first research question as to what extent students acquire a more differentiated understanding by working with personal narratives compared to factual texts—not only is the number of reasons given crucial, but the number of misconceptions identified can also indicate an incorrect contextualisation of the factual knowledge (sub-question 1.3.). We, therefore, compared the average number of misconceptions from the pre-tests with the average number of misconceptions from the first part of the writing task (the list of key points). We then determined the average number of misconceptions from the second part of the writing task (Karim’s self-talk) and compared the results of both groups.

Figure 3 shows that the number of misconceptions in the first part of the writing task increased by 300% for group B (who had worked with the factual text), from an average of 0.47 in the pre-test to 1.88 in the list of key points. However, in group A (who had worked with the personal narratives), the increase was only about 25%, from an average of 0.63 to 0.79. We assume this marked difference is due to the nature of the text material given to the students. It was observed that the misconceptions in the pre-tests could mostly be attributed to a lack of knowledge about the living conditions in Egypt. However, the majority of the misconceptions that were formed after reading the text result from the generalisation of individual situations. 

As an example, the first two text examples from the pre-test and the subsequent two comparative examples from part 1 of the writing task are given: 

Example 1 (pre-test):

*“They leave their village because they don’t have enough drinking water there or hardly any shade.”*

(Code RI 30)

Example 2 (pre-test):

*“They leave their village because Cairo is planted. Egypt is mostly desert and very warm during the day. Also, a desert can be dangerous and buildings can be destroyed by a sandstorm. In the desert it is often hard to find water or food, whereas in Cairo you have access to water and planting of fruits is possible.”*

(Code CE 16)

Both students imagined an Egyptian village in the middle of the desert without shade, at risk of sandstorms and lacking water and food. The natural geographic conditions in the fertile Nile Delta are unknown to them. Cairo appears comparatively rich in water and fertile soil and, thus, must be attractive to desert dwellers. This kind of misconception no longer occurred after reading the text. Instead, there were inaccurate generalisations, as the following two examples from the list of key points show:

Example 3 (list of key points written after reading the text):

*“(a) earn extra money for the family*

*(b) build your own life*

*(c) search for work*

*(d) study at technical colleges and universities*

*(e) better paid work because there is too much competition in agriculture, such as large agricultural companies, which produce cheaper*

*(f) better air quality*

*(g) more space between the residential buildings, wide streets and well-maintained green areas.*

*(h) modern shopping centers*

*(i) various sports facilities.”*

(Code CH 108, compiled on the basis of the factual text)

Example 4 (list of key points written after reading the text):

*“(a) dream of big money*

*(b) more work options*

*(c) running electricity and water* (sic!)

*(d) feeding the family*

*(e) high reputation”*

(Code LO 26, compiled on the basis of the factual text)

In example 3, the student first gave five appropriate reasons (a–e). However, reasons f, g, h and i only applied to certain districts of Cairo and were described in the text material in connection with the living conditions in one of the new, modern satellite towns of Cairo. The living conditions in the poor neighbourhoods, also described in the text material, were not mentioned by the student. In most residential areas of Cairo, for example, the air quality is considerably worse than in the countryside. Shopping malls and sports facilities cannot be considered as realistic reasons for internal migration from rural areas either, as most of the migrants cannot afford them. A similar, also inaccurate, generalisation can be found in example 4: While aspects a, b, d and e adequately reflect the reasons of most migrants, c must be counted among the misconceptions. Electricity and running water are not available everywhere in the capital, but they are in most rural areas. This information is also included in the text material. 

In the next step, we examined the misconceptions in part 2 of the writing task (Karim’s self-talk). The average number of misconceptions in group A was 0.26, whilst in group B it was 0.71 (Figure 3). This indicates that the number of misconceptions in group A was almost two thirds (63%) lower than in group B. However, it must be added that misconceptions only make up a small part of the reasons used in both groups. 

In summary, the students’ texts written on the basis of the personal narratives were more detailed with regard to the reasons given for or against Karim’s move to Cairo and had fewer misconceptions than the students’ texts written on the basis of the factual text. Sub-question 1.3 can, therefore, be answered in the affirmative: the students’ texts showed clear differences in content between the two groups of students, which indicates that the personal narratives enabled our test persons to have a more differentiated understanding of the topic than the factual text. 

#### 4.1.4. Sources of the Reasons Used for or against Internal Migration

Question 1.4. asked about the significance of the text material for the content-related differences in “Karim’s self-talk” between the two groups. The text material includes the personal narratives and the factual text, the introductory text to the writing task, which is identical for both groups, and the students’ independently developed ideas. By analysing the students’ texts, we wanted to find out which of the text material was predominantly used and whether there were any clear differences between the two groups. Furthermore, we were interested in whether the students working with the personal narratives transferred the knowledge gained more creatively and appropriately to the life situation of the 17-year-old Egyptian Karim than the students working with the factual text.

**Figure 4:**
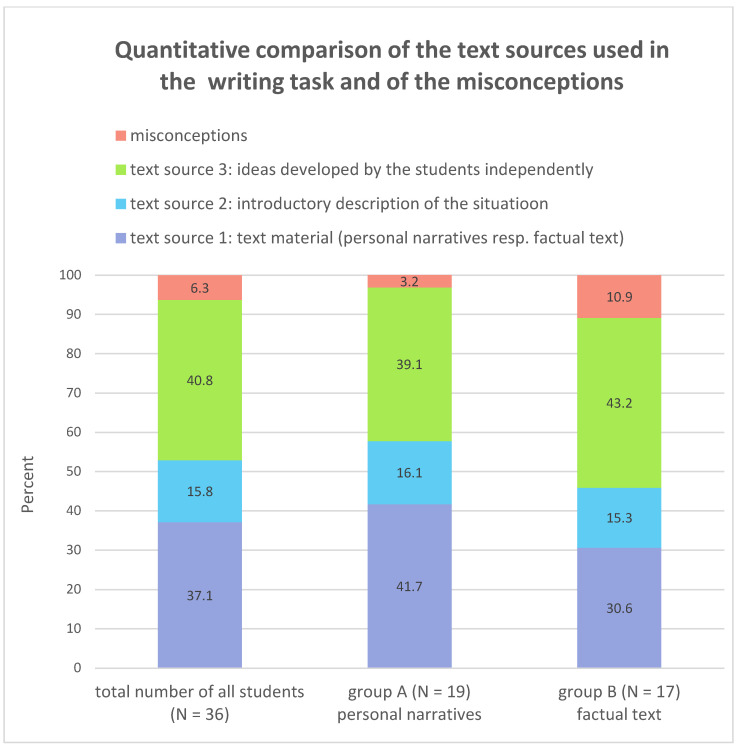
Quantitative comparison of the text sources used in the writing task and of the misconceptions (authors’ diagram).

Figure 4 illustrates the results. All three text sources were used, but to a varying extent. In the 36 student texts, more than half (52.9%) of the appropriate reasons were taken from the given material and 40.8% of the appropriate reasons were developed by the students themselves, in which their own life experiences and ideas of life were transferred coherently to the life of 17-year-old Karim in Egypt. The high proportion of independently developed reasons was remarkable. Since the percentages of the two groups do not differ significantly (39.1% and 43.2%, respectively), the reason for the variation cannot lie in the different text material given. We assume that the reason lies in the nature of the writing task. Formulating a self-talk is not possible without developing empathy with the life situation of the person concerned. The success in empathising with the life circumstances of 17-year-old Karim was thought to be due to the fact that the 14- to 15-year-old students belong to roughly the same age group as Karim and live in a rural area themselves. For most of them, the same question will arise after leaving school as for Karim: stay in the village they grew up in or move to a big city to start training or studying or find a good job? The pros and cons of moving to the city are certainly familiar to the students through older siblings and friends and as such they can convincingly present the relevant arguments in the context of the self-talk.

Figure 4 also shows quantitative differences between group A and group B in terms of the frequency of use of the text material that was given (text source 1 and text source 2). Source 1 is different for both groups and includes the personal narratives and the factual text. However, source 2 is identical for both groups and consists of the introductory description of the writing task, in which Karim’s living conditions are vividly described. It is noted that group A used their text source 1, the personal narratives, for 41.7% of Karim’s argumentation in the writing task. Group B, on the other hand, used their text source 1, the factual text, for only 30.6%. In contrast, text source 2 was hardly used at all (16.1% and 15.3% respectively). It seems reasonable to assume that the personal narratives are better suited for the writing task than the factual text due to their vividness and closeness to everyday life. 

Sub-question 1.4.—to identify whether there are clear differences between the two groups in the use of the given text material—has a less clear answer than previous questions. On the one hand, the personal narratives do not seem to create better empathy with the situation of the young Egyptian migrant compared to the factual text. On the other hand, when identifying a list of reasons, the personal narratives are used more extensively than the factual text.

### 4.2. Memory

To answer the second research question—whether knowledge is better remembered when acquired through personal narratives instead of a factual text—we compared the first part of the writing task (list of reasons for internal migration to Cairo) with the results of the post-test on the same question. The post-test took place three weeks after the writing task. Figure 3 illustrates the results in columns 2 and 4 for both groups. The data show that, in both groups, the difference in the documented level of knowledge between the first part of the writing task and the post-test is extremely small. The knowledge acquired through reading the text material was still very well remembered three weeks later, regardless of the type of text material. For group A, the average number of reasons given after reading the text was 2.74, whilst it was 2.88 in the post-test. the corresponding values for group B were 2.35 and 2.29. The second research question can, therefore, be answered in the negative: personal narratives did not have a more positive effect on the recall ability of our test persons compared to factual texts. 

### 4.3. Motivation

The third part of the school study analysis refers to the students’ motivation regarding their work with the provided text material. For this purpose, we evaluated the polarity table and written comments of the students, which they had written immediately after completing the writing task. The guiding question was whether the students who had worked with the personal narratives gave more positive feedback than the group who had worked with the factual text. If this question can be answered in the affirmative, it is likely that the personal narratives have a more motivating effect on the students and their work than the factual text. 

#### 4.3.1. Evaluation of the Polarity Table

**Figure 5:**
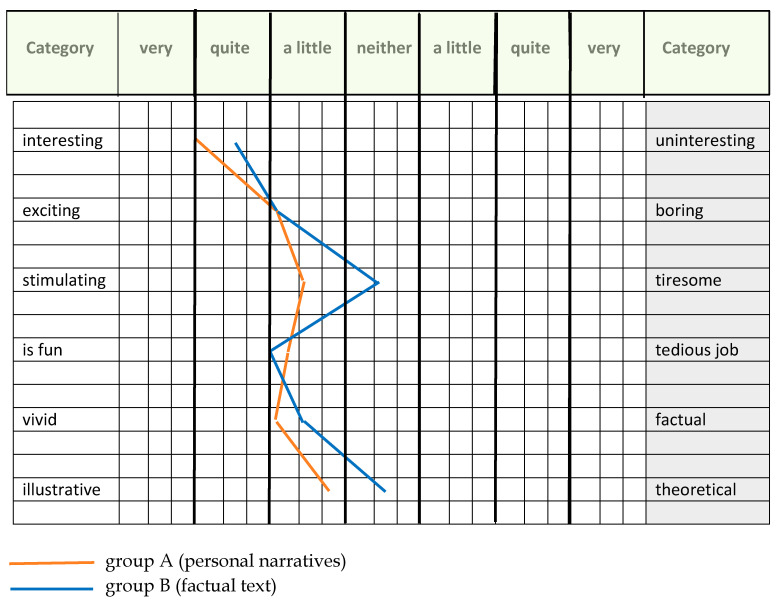
Polarity profile for the assessment of the text material by the students according to six predefined categories (modified order and arrangement, authors’ diagram).

The results of the evaluation of the polarity table are visualised in Figure 5 and show that there are marginal differences between the scores of groups A and B for most pairs of polarities. Since the sample number is small—20 participants in group A and 17 in group B—these small differences cannot be considered meaningful. However, there is a clear difference in the scores for the pair “stimulating/tiring”. We consider this striking difference to be significant with regard to the research question. Personal narratives are perceived as stimulating and it can be assumed that they have a beneficial effect on work motivation. Factual texts, however, have a tiring effect on many students and may, therefore, have the opposite effect in terms of work motivation. 

#### 4.3.2. Evaluation of the Students’ Comments

Furthermore, the students had the option to provide a personal comment on their work with the text material. In both groups, this option was used by the majority of the participants. The comments relate to the content and length of the text material, its relevance to their own lives, the writing task and the lesson of the school study in general.

##### Content of the Text Material

The comments from group A unanimously rated the content of the text material as positive, with regard to the possibility to get to know the individual experiences of other people through the personal narratives and, thus, put oneself in their shoes:

*“I found the texts of the people from Egypt very interesting and different from what one usually hears. They usually only talk about advantages and disadvantages and not about how people who have experienced it directly report it. That’s what I liked most about these texts, that is, people’s personal experiences.”*

(Group A, Code EL 56)

In contrast, the content-related comments of group B emphasised the clear presentation of the advantages and disadvantages of living in rural areas or in Cairo:

*“Through the text, the situation of the city and the countryside was brought closer to me once again. Advantages and disadvantages were good to find out.”*

(Group B, Code MI 24)

##### Length of the Text Material

It is noticeable that the length of the text is mentioned more frequently in group A than in group B. A third of the students in group A commented on text length and unanimously complained that the texts were too long:

*“Was a lot of fun and really cool, except there was too much text.”*

(Group A, Code NE 24)

On the other hand, the text length was mentioned negatively only twice in group B. The factual text with 949 words was obviously adequately designed for the majority of the students, while the personal narratives with 1590 words were a very big challenge for one third. 

##### Personal Relevance of the Text Content

The personal relevance of the text contents was only reflected upon by one student from group A:

*“The survey/texts have made me realise even more how well I am doing. And that I should use my future, because (erg. other) people will never be able to do this properly, because they simply don’t have the financial means. And they therefore have to do what the family asks of them forever. They can never realise their goals properly.” *

(Group A, Code LU 9)

##### Assignment of the Writing Task

The actual task was only commented on by students in group B. On the one hand, this was done objectively:

*“The tasks were understandable and reasonably interesting”*

(Group B, Code EL 27)

but on the other hand, was also empathetic:

*“I enjoyed writing a continuation of his (Karim’s) thoughts because while reading I had many ideas that I applied in writing.”*

(Group B, Code CE 16)

##### Lesson Unit of the School Study

The last category of comments was concerned with general statements about the lesson of the school study. The judgements were consistently positive and emphasised “the different” compared to the usual lessons. This assessment was concluded by more than half of the students from group A and in just under one third of the students from group B.

*“I think this way of learning is much better than opening the book in class, being told a page, working on it and getting the feeling that the teacher doesn’t care. I think it is well done.”*

(Group A, code JU 10)

*“I found the text relatively interesting. It was something different.”*

(Group B, Code JA 24)

One commentary also reflected on the atmosphere of the lesson:

*“In my opinion it was very interesting and a relaxed work. The fact that everyone was busy made it a very pleasant lesson.”*

(Group A, Code RI 30)

With regard to the third research question, the results can be summarised as follows: On the one hand, both groups evaluated their work material as predominantly positive, but on the other hand, the students also consciously reflected on the different ways of experiencing the topic. Group A emphasised the possibility of perceiving the topic from the perspective of those affected as positive, while group B mentioned the easily understandable comparison of advantages and disadvantages. Against the background of a didactic approach that not only wants to impart knowledge but also enables a change of perspective, this reaction of the students confirms the suitability of the personal narratives in this respect. However, it must be taken into account that not all students are able to read the text material without difficulty. A careful selection with regard to the learners’ abilities is, therefore, essential if motivation is not to be impaired.

## 5. Discussion

The intention of this article was to theoretically demonstrate the subject-related and didactic potentials of authentic personal narratives for geography lessons, and to empirically test them in comparison with factual texts. If we understand geography lessons as a “place of questioning”, whereby “every question requires insightful and profound thinking” to understand the topic concerned [7] (p. 45), then we need a teaching design that enables questioning. Authentic text media, vividly narrated from a personal perspective, can contribute to this because:

- Authentic personal narratives verbalise personal experiences of the world and, thus, point out that, according to constructivist epistemology, every experience of the world is subject-bound and, thus, must be interpreted against the background of its context of origin [27];
- Authentic personal narratives express emotions that can be regarded as a constitutive part of world experience and as such must be included in the process of interpretation in a reflective way [29,30];
- Authentic personal narratives describe actions or intentions of action that make it clear that spaces can only be understood with the inclusion of the people acting in them and thereby shaping them [35,36,37].

Personal experiences of the world, the emotions associated with them and the resulting intentions for action require questioning and interpretation within the context of their origin. They are not the "absolute truth", but perspectives in need of reflection. In this respect, they differ fundamentally from textbook texts that convey their contents in such a way that they appear as "objective truth", although these texts are also perspective-bound and shaped by the textbook authors’ understanding of the world and the subject [46].

Within the framework of a school study with 14- to 15-year-old students from a grammar school in Germany, we investigated the results of working with personal narratives in comparison to working with a factual text. The areas of investigation were the students’ understanding of the content of a geographical topic, their memory performance and their motivation to work. The topic chosen was internal migration in Egypt, which the participants of the study had to work on in the form of a creative writing task. 

With regard to content-related understanding, we wanted to find out to what extent students can acquire a more differentiated understanding of geographical topics by working with personal narratives compared to working with factual texts (research question 1). The evaluation of the school study revealed that both types of texts are equally suitable for conveying and acquiring factual knowledge. However, beyond the acquisition of knowledge, the personal narratives enabled a more multi-layered and, thus, deeper understanding of internal migration in Egypt, as the results of the creative writing task showed. As part of this task, the students were asked to formulate the advantages and disadvantages of moving to Cairo in the form of a self-talk from the perspective of a young person from a rural region of Egypt. The “self-talks” of the students who worked with the personal narratives showed significantly fewer misconceptions than the texts of the comparison group (Section 4.1). This difference made sense when one took a closer look at the nature of the misconceptions.

The majority of the misconceptions that occurred when working with the factual text were based on an inappropriate contextualisation of the newly acquired factual knowledge. The diversity of the aspects of internal migration presented in the factual text was probably only partially perceived by the students. In their “self-talk”, they therefore generalised individual aspects into an overall picture that largely lacked inner differentiation. Personal narratives, on the other hand, already make it clear through their text format—several short texts by different authors—that different aspects and/or points of view are involved. In this respect, they facilitate and support the process of consciously considering and perceiving multiple perspectives. 

In addition, personal narratives have a different effect on their readers when compared to factual texts due to their specific linguistic style [25] (pp. 264–273). In narration, an experience is expressed in a personal and emotional, and sometimes exciting, way. The recipients can be addressed directly and possibly even be included in the narrative. This can facilitate empathy with other perspectives and deepen their understanding. Empathy, in turn, is the initial point of any change of perspective, because “being able to put oneself in another person’s shoes is the prerequisite for understanding them and seeing the world through their eyes” [32] (p. 14). The result of the creative writing task confirms this in a convincing way: the students who had worked with the personal narratives took into account a significantly greater variety of potential developments regarding the acceptance of their decision in the family, professional success or failure in Cairo or their personal future (starting a family, returning to the village, etc.) in their “self-talk” answers. 

A possible negative of personal narratives is that they only express subjective approaches to the world and are, therefore, unsuitable for a comprehensive understanding [25] (pp. 266f), but this can be refuted with regard to the didactic function of these narrative texts in the context of geography lessons. A personal narrative forms the *beginning* of the examination of a complex topic. It must subsequently be relativised in its significance through critical contextualisation work and supplemented by further texts or materials. Only then can a differentiated, multi-perspective picture of reality emerge [27,47].

With regard to memory performance, we were interested in the extent to which geographical content is better remembered when being conveyed through personal narratives compared to a factual text (research question 2). The results of the school study show no difference in recall performance between the two groups (Section 4.2). Thus, the personal narratives did not have a more favourable effect on the recall of the contents compared to the factual text. This result surprised us, because it contradicts the generally accepted research findings in psychology on the connection between emotions and memory performance [40,48,49]. One possible explanation is that the personal narratives used in the study did not express emotions as a priority, and were hidden between the narrated facts: “It is *not a nice* place, but when I have my children with me *I feel safe*” (source text 4), “*unfortunately* the villas were demolished later” (source text 5), “it is *too dangerous* there for an unmarried young woman” (source text 7). This result should be seen as a suggestion to make the emotional part of the narrative more clear for the students’ field of perception, by selecting a text in which the emotional encounter with the world is more prominent, for example. 

With regard to the learners’ motivation to work, we investigated whether the students were more motivated when they worked with personal narratives compared to working with factual texts (research question 3). For this purpose, we evaluated a polarity table, in which the students were asked to assess the texts used according to given criteria, as well as the students’ personal comments (Section 4.3). In their comments, the students who had worked with the personal narratives point out that they were encouraged to reflect on their own economic and social living conditions. They also appreciated the possibility of being able to participate in the individual experiences of other people, as the personal narratives enabled them to put themselves in the shoes of these people. These statements from the subjective point of view of the students, thus, confirm the potential of personal narratives, which has already been theoretically substantiated, making them aware of the subject-bound nature of any perception of the world and the associated invitation to reflect on the position of others and one’s own. They also confirm their potential to empathise better with other people and to understand their actions in space through a change in perspective.

Another interesting result of the school study concerns the students’ reaction to the task of writing a creative text in the form of a reflective self-talk from the perspective of an Egyptian youth. The feedback highlighted the individualised nature of the writing task as positive and motivating. This can be understood as a call to not only reserve creative writing for language teaching, but also to integrate it into subject teaching more frequently. Research on the importance of writing in geography lessons has shown that there are still clear deficits in this direction [50].

## 6. Conclusions

The essentially positive feedback from the students on working with personal narratives supports our concern to demand greater use of authentic personal narratives in geography lessons. The presented professional and didactic potentials of this type of text, as well the observed acceptance by the students, clearly speak in favour of it. 

Special attention must be paid to the selection of the narrative texts, which should not be random. The selection should strive to reflect the perspectives that exist in reality as representatively as possible, even if this can ultimately only be implemented approximately [27,44,51,52]. Digital media, along with print media and books, are a valuable resource for obtaining authentic texts. Care should be taken that personal narratives are not “dressed up factual texts”, but rather vivid narratives that also reflect personal emotional experiences. In the selection, the abilities of the target group should be taken into account with regard to the length of the texts and their level of difficulty, so that neither are over- or under-challenging learners’ limits and their motivation to work. Personal narratives with varying degrees of difficulty can be used profitably, especially in teaching classes with a wide range of students’ abilities. 

Personal narratives can also be used very well with regard to competence-oriented teaching. The acquisition of subject competence is combined with methodological competences (reflection on perspectivity and contextualisation), argumentation competence (reasoned assessment and evaluation of text statements) and social competences (internally differentiated and/or cooperative working methods). In view of the rapidly increasing possibilities to obtain information by mouse click and to have complex reasoning contexts written by the use of artificial intelligence, the targeted development of independent thinking and judgement activities in class is an urgent task. In addition, personal narratives offer numerous possibilities to include creative tasks in lessons.

Personal narratives still represent a broad field for future research. With regard to the targeted use of the texts in class, it is certainly worthwhile to research the effect of different types of narration on learners. The same applies to their suitability with regard to the development of argumentation skills [53,54,55]. Additionally, a fruitful research topic can still emerge from the various possibilities of combining personal source texts with creative work assignments. 

In his bestselling book “The Empathic Civilization”, sociologist Jeremy Rifkin formed a perspective on the development of human society that focused on the empathic capacity of human beings. “The global public square is fast becoming a reality (…). We are within reach of thinking of the human race as an extended family—for the very first time in history (…)” [56] (p. 443). By engaging with the authentically other in their personal way of encountering the world, geography lessons can also contribute to the development of empathy and understanding of others. 

## Figures and Tables

**Table 1:** Table of the personal narratives used in the school study and their selection criteria.

	Criterion 1: Academic Perspective	Criterion 2: Social Groups (Formed from the Following Four Criteria)				Criterion 3: Individual Perspective
		Place of Residence	Age Group	Gender	Social Class	
**Text 1**	economy, living together in society	village in the Nile Delta	retired 58 y.	male	middle class	advantages of village life in retirement
**Text 2**	education	village in the Nile Delta	student 20 y.	male	lower class	studying in Cairo
**Text 3**	economy	village in the Nile Delta	working approx. 45 y.	male	lower class	competition from agribusiness
**Text 4**	economy, living together in society	village in Upper Egypt	working approx. 30 y.	female	below the mini-mum subsistence level	family reunion in Cairo
**Text 5**	urban development, transport	affluent inner-city district of Cairo	retired 80 y.	female	upper class	a look into Cairo’s past
**Text 6**	urban development, transport, living together in society	affluent neighbourhood in a satellite city of Cairo	working 47 y.	male	middle class	advantages of living in the new desert cities
**Text 7**	living together in society	village in an oasis	working approx. 50 y.	female	lower class	a look at the dark side of city life

**Table 2:** Text example from the writing task, categorised by reasons and text sources.

Text Written by a Student (Code: EM 76)	Categories for Reasons for Migration	Text Source of the Reasons
*Today I thought again whether I should stay here in the village or whether I would rather go to Cairo.*	introductory sentence	writing task
*Hani, one of my best friends, thinks I should do it,*	reason 1 (for the move): advice from a friend	introductory text for the writing task
*because I study at a university there*	reason 2 (for the move): possibility of studying	text material
*and can then look for a much better-paid job.*	reason 3 (for the move): prospect of better paid work in Cairo	text material
*After a few years, I could also bring my family to Cairo.*	reason 4 (for the move): possibility of family reunification in Cairo	text material
*But it also becomes a problem to rent or buy a suitable flat in Cairo, especially because of the prices and people.*	reason 5 (against the move): housing problem in Cairo	text material
*Another friend of mine thinks so too and advises me against moving there.*	reason 6 (against the move): advice from a friend	introductory text for the writing task
*I think he’s even right; since I don’t own a car, I always have to take the bus, which also costs money.*	reason 7 (against the move): the problem of getting around in Cairo	independently developed ideas
*I think I’ll consider moving there in a few years when I have a bit more money.*	final decision	independent consideration

**Table 3:** Polarity table of the school study for the evaluation of the text material (modified order).

No.	Category	Very	Quite	A Little	Neither	A Little	Quite	Very	Category
**1**	interesting	o	o	o	o	o	o	o	uninteresting
**2**	exciting	o	o	o	o	o	o	o	boring
**3**	stimulating	o	o	o	o	o	o	o	tiresome
**4**	is fun	o	o	o	o	o	o	o	tedious job
**5**	vivid	o	o	o	o	o	o	o	factual
**6**	illustrative	o	o	o	o	o	o	o	theoretical

## Data Availability

The data presented in this study are available on request from the corresponding author. The data are not publicly available due to data protection and privacy.

## Supplementary Materials

The following supporting information can be downloaded at: https://www.mdpi.com/article/10.3390/ejihpe13060081/s1, Supplementary: Factual text.
