# Supplementary material for: “It’s Easy to Put Oneself in the Shoes of Others.” Results of a School Study in Geography Lessons on Working with Authentic Personal Narratives in Comparison to Factual Texts
Source: Eur J Investig Health Psychol Educ. 2023 Jun 18;13(6):1058–81. doi: 10.3390/ejihpe13060081 (PMC10296907; doi:10.3390/ejihpe13060081)
Supplement: Supplementary file 1 [file ejihpe-13-00081-s001.zip › ejihpe-2320472-supplementary.pdf]

European Journal of
Investigation in Health, Psychology and Education

Supplementary: Factual text

"It's Easy to Put Oneself in the Shoes of Others." Results of a School Study in Geography Lessons on Working with Authentic Personal Narratives in Comparison to Factual Texts

Urbanisation is a global process. The term "urbanisation" refers to the fact that more and more people in many countries are living in cities. In 1960, 33% of the world's population lived in cities; by 2020, this figure had risen to 56%, or more than half. But there are big differences between countries. In Belgium, for example, 98% of all people live in cities, in Japan it is 94%. In Papua New Guinea and Burundi, on the other hand, only 13% of the population live in cities. In Germany, the urbanisation rate is 76%.

Increasing urbanisation means that more and more villages have enough inhabitants to be called cities. The number of cities is, therefore, increasing. Urbanisation is also increasing the population of large cities. Megacities such as Tokyo, Shanghai, Mexico City and New York with more than 10 million inhabitants are emerging. In Europe, megacities include Moscow, Istanbul, Paris and London. The Rhine-Ruhr conurbation also has more than 10 million inhabitants. As the population grows, cities need to build more and higher housing. As a result, urban development is becoming denser and the urban area larger. Where only a few decades ago the outskirts of cities were farmland and villages, today there are new industrial estates, housing estates, business districts and motorways into the city centre.

Although urbanisation processes are taking place almost everywhere in the world, there are specific features in each country that result from different living conditions. The following section takes a closer look at urbanisation in Egypt. In 2020, Egypt had a population of about 102 million, of which about 44 million lived in cities. The degree of urbanisation was, therefore, 43%. The capital Cairo is particularly affected by urbanisation.

Cairo is currently home to around 20 million people, or 20% of all Egyptians (by way of comparison, Berlin is home to just 4.2% of the total population of Germany). Strictly speaking, Cairo is an agglomeration of three large cities: Cairo, Giza and Qalyubiya. The urban area also includes several smaller cities that have been built in the surrounding desert in recent decades. The three major cities and these desert towns are collectively referred to as "Greater Cairo".

The population of Greater Cairo is growing because every year more children are born than old people die. This is known as natural population growth. There is also a certain number of people who move to the capital region from other villages or towns in Egypt. This is called internal migration. Mostly, it is younger men who leave their homes to look for work in the capital. When they find work, they sometimes bring their families with them. Others come to study at one of the many technical colleges and universities.

Life in Egypt is not easy, especially for farmers. Because the climate is always warm, they can grow grains, vegetables and fruit all year round and harvest two or three times a year. But when they try to sell their produce to grocery stores, they find it hard to compete with large agricultural companies that can offer their goods at lower prices. As a result, some farmers sell their land and look for new, better paid work in the city.

Life in the megacity of Cairo is different from life in a village in many ways. Firstly, it is not easy for people from the countryside to find suitable accommodation. In the villages, they live in their own houses, but in the city, they have to rent or buy an apartment. Rich Egyptians like to buy apartments and houses in one of the newly built cities in the desert. These new desert towns are easy to reach by car from Cairo's city centre on multi-lane motorways. Depending on the distance and time of day, the journey takes between

half an hour and two hours. In the desert cities, the air quality is better and there is more space between houses, with wide streets and well-maintained green areas. Modern shopping centres cater for daily and medium-term needs, with cafés and restaurants. Sports enthusiasts will find a wide range of different sports facilities. However, the public transport system is often unsatisfactory. Those who do not own a car usually have to rely on taxis. Public buses do not serve all parts of the desert cities. Some residential areas are fenced and guarded, known as "gated communities". Because the desert cities are attractive to the wealthy, property values increase many times over in a few years.

Living conditions in downtown Cairo vary greatly from neighbourhood to neighbourhood. In some neighbourhoods, there are still beautiful old villas with large gardens that have been in family ownership for many decades. However, it is not uncommon for residents to move into more modern houses in the new desert cities. The old villas then fall into disrepair and are eventually demolished and replaced by multi-storey buildings to accommodate the growing population. As a result, people are living closer and closer together, and the roads are becoming increasingly congested with cars, causing daily traffic jams and serious air pollution. The close proximity of people also leads to social control, which on the one hand creates a sense of security, but on the other hand significantly reduces privacy. People can easily observe each other and learn many things that should be private. On the other hand, a stranger is easy to spot, which makes it much harder for criminals.

Some areas on the outskirts of Cairo can be described as slums. There, families live in extreme poverty and cramped living conditions with no running water or electricity. Their income is so low that they can barely afford to live. Everyone in the family has to help earn money, including the children.

(Note: The original version of this text was presented to the participants of the school study in German).
